# False positive antigen test for *Dirofilaria immitis* after heat treatment of the blood sample in a microfilaremic dog infected with *Acanthocheilonema dracunculoides*

**DOI:** 10.1186/s13071-020-04376-9

**Published:** 2020-10-01

**Authors:** Viktor Szatmári, Martin Willem van Leeuwen, Christine Jantine Piek, Luigi Venco

**Affiliations:** 1 Department of Clinical Sciences, Faculty of Veterinary Medicine, Utrecht University, Yalelaan 108, 3584 CM Utrecht, The Netherlands; 2 Clinica Veterinaria Lago Maggiore, Corso Camillo Benso Cavour, 3, 28040 Dormelletto, NO Italy

**Keywords:** *Acanthocheilonema*, Cross reactivity, *Dipetalonema*, Helminth, *Hepatozoon*, Moxidectin, Nematode, Parasite, Patent ductus arteriosus, Tick

## Abstract

**Background:**

*Dirofilaria immitis* is responsible for heartworm disease in dogs in endemic areas worldwide. Screening for this infection is done by blood tests. Antigen testing is the most sensitive method to detect an infection with adult (female) worms. Microscopic examination of a blood smear or Knott’s test can be used to detect circulating microfilariae, the infective larvae. To increase the sensitivity of the antigen test by decreasing the false negative test results, heating of the blood sample has been recommended in recent guidelines. Heating is believed to remove blocking immune-complexes. Circulating microfilariae are not specific findings for heartworm infection, as other nematodes (among others, *Acanthocheilonema dracunculoides*) can also result in microfilaremia. Although the type of microfilariae cannot be determined by microscopy alone, real-time PCR can reliably identify the infecting nematode species. Correct identification of the parasite is of major importance, as an infection with *D. immitis* requires antiparasitic therapy, whereas *A. dracunculoides* is thought to be a clinically irrelevant coincidental finding. The present case report describes a microfilaremic dog where the initial antigen test for *D. immitis* turned positive after heat treatment, whereas real-time PCR revealed that the microfilariae were *A. dracunculoides* (syn. *Dipetalonema dracunculoides*).

**Results:**

A circa 5-year old, asymptomatic Spanish mastiff dog was referred for heartworm therapy because microfilariae were found via a screening blood test. The dog was recently imported to the Netherlands from Spain, where it had been a stray dog. Antigen tests on a plasma sample for *D. immitis* were performed with three different test kits, which all turned out to be negative. However, heat treatment of two of these samples were carried out and both of them led to a positive antigen test result. Real-time PCR showed that the circulating microfilariae belonged to *A. dracunculoides* species. Three administrations of moxidectin spot-on at monthly intervals resulted in a negative antigen and a negative Knott’s tests one month after the last treatment.

**Conclusions:**

We conclude that heat treatment of initially negative blood samples for *D. immitis* could lead to false positive antigen test results if the dog is infected with *A. dracunculoides.*
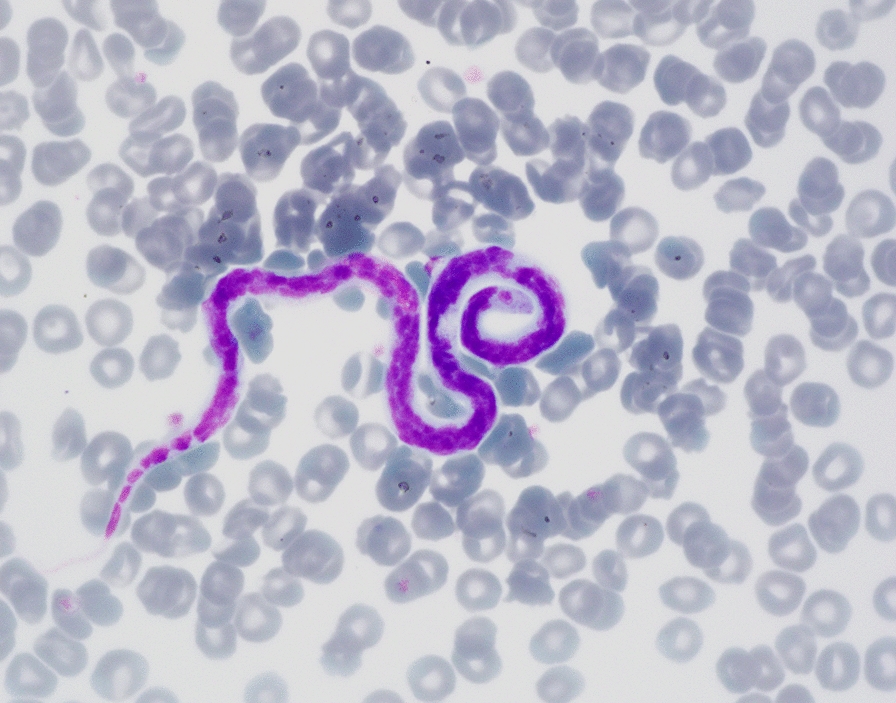

## Background

*Dirofilaria immitis* is responsible for heartworm disease in various pet species such as dogs, cats and ferrets, and it is a clinically relevant canine parasite in endemic areas all over the world [1]. Antigen testing on a blood sample is the most widespread used method to screen dogs for the presence of adult worms [1–3]. The test is an ELISA immunoassay that detects the circulating antigens released by the reproductive tract of the adult female worms [1–3]. False positive test results due to potential cross-reactions with both natural and experimental infections with *Angiostrongylus vasorum*, *Acantocheilonema reconditum* and *Spirocerca lupi* have been reported in dogs, while others have described cross-reactivity with *Acanthocheilonema odendhali* in sea lions [4–7]. Recent studies in dogs showed that heat treatment of initially negatively tested plasma samples can increase the sensitivity of ELISA immunoassay, reducing the number of false negative test results [8]. False negative test results arise from blocking immune-complexes, which heat treatment can resolve. However, false positive antigen test results have been reported after heat treatment of the blood samples in dogs that were infected with *A. vasorum* and *D. repens* [9, 10].

If adult male and female heartworms are present in the same canine host, circulating microfilariae will be produced, which can be detected with microscopic examination of a native blood smear or with Knott’s test. Microfilariae are not specific for heartworm infection, as infection with several other nematodes can also result in microfilaremia, such as *D. repens*, *A. dracunculoides* and *A. reconditum* [11–15]. Though cytological differentiation of microfilariae from *D. immitis* and *D. repens* is possible (as the head of *D. immitis* contains no nucleoli, whereas *D. repens* has two sub-terminal nucloeli in their heads), differentiating the microfilariae of *D. immitis* from *A. dracunculoides* is not possible, as the heads of both microfilaria species lack nucleoli [12]. Though the microfilariae of the genus *Acanthocheilonema* are significantly shorter than those of the genus *Dirofilaria*, their morphometric identification is in practice unreliable [13, 14]. Recently, commercial veterinary laboratories offer the possibility of highly specific molecular diagnostic techniques (real-time PCR, IDEXX Europe B.V. Hoofddorp, the Netherlands) for identification of microfilariae. This test is especially useful in cases where the antigen test for *D. immitis* is negative in the presence of circulating microfilariae.

Adulticide therapy of heartworm infected dogs is recommended by the current guidelines of the American Heartworm Society, even if the positive tested dogs show no clinical symptoms [1]. This means, that asymptomatic dogs with a positive antigen test due to a subclinical infection are recommended to be treated with an adulticide protocol too. In these mildly infected dogs, additional diagnostic tests, such as thoracic radiographs and echocardiography typically show no abnormalities, therefore these tests are not helpful in confirming or ruling out an infection with *D. immitis*, when screening blood test results are equivocal. A high specificity of the blood antigen test is therefore of major importance. Adulticide therapy for dogs with heartworm infection using the currently recommended protocol of the American Heartworm Society with melarsomine is not only expensive, but the administration of melarsomine means multiple painful intramuscular injections followed by two months of cage rest, both of which contribute to a negative impact on the quality of life of the patient [1]. For these reasons it is important to keep the number of false positive screening test results as low as possible.

## Methods

### Case presentation

An estimated 5 year-old, 47 kg, neutered female Spanish mastiff dog was presented to the cardiology service of the authors’ institution for treatment of a suspected heartworm infection based on a recent detection of circulating microfilariae via a screening blood test performed by the referring veterinarian. The testing was performed as the dog had been a stray in Spain and was imported into the Netherlands one month earlier. The owner noticed no abnormalities in the general functioning of the dog; however, following every walk, a warm swelling of the left hind leg between the tarsal and the stifle joint appeared within an hour’s time, and subsequently the dog stopped walking. The swelling disappeared spontaneously every time by the next day. Two months before import, the dog had tested positive for *Leishmania infantum* (titer 1:640) and for *Anaplasma canis*, and negative for *Erhlichia canis* antibodies and for *D. immitis* antigens via a blood examination in the animal shelter, where it had been living for about 6 months. For the leishmaniasis daily oral allopurinol therapy was started (4.8 mg/kg BID).

**Fig. 1:**
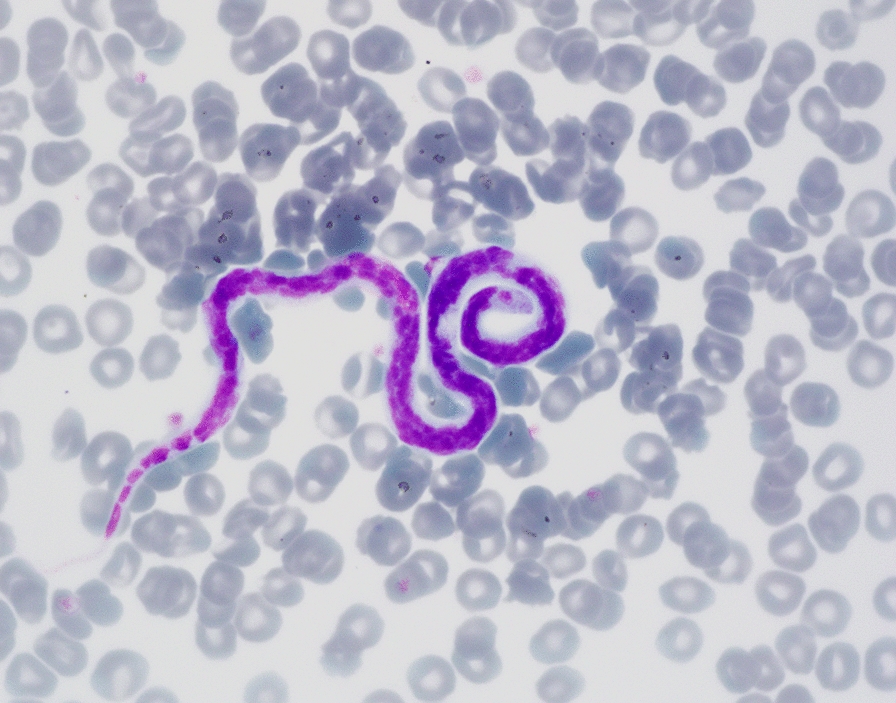
Photomicrograph of a microfilaria of *Acanthocheilonema dracunculoides* in a blood smear of a dog. The central body of the microfilaria is filled with dense nuclei, while the cephalic and caudal ends are nuclei-free. Because the same microscopic features can be recognized in microfilariae of *Dirofilaria immitis*, they are indistinguishable in a blood smear. May-Grünwald & Giemsa staining, 1000 × magnification

An additional blood examination was performed at the laboratory of the authors’ institution. Routine hematology and biochemical blood work revealed no abnormalities, except for a mild hyperproteinemia (8.9 g/dl; reference 5.2–7.6 g/dl) due to a mild hyperglobulinemia. Besides many microfilariae (Fig. 1), a blood smear also revealed *Hepatozoon* gamonts. Based on microscopic examination of 400 neutrophil granulocytes, 0.5% of them contained gamonts. Serological examination for *Leishmania* was positive with a titer of 1:5120. An antigen test (FASTest® HW antigen test-kit, Diagnostik MEGACOR, Hörbranz, Austria) for circulating antigens of *D. immitis* was negative, but after heat treatment of the blood sample the test turned positive. The heat treatment was performed by keeping the plasma sample in a 100 °C water bath for 10 min, then centrifuging the sample for 5 min at 12,000 × *rpm*, and finally repeating the antigen test from the supernatant [8].

The referring veterinarian performed radiographs of the left hind leg, which were interpreted by a European veterinary specialist in diagnostic imaging of the authors’ institution. Radiographic examination showed a large amount of irregular new bone formation in the distal third of the tibia with marked thinning of the cortex and a mild to moderate osteolysis with a large zone of transition. The new bone formation in the mid to proximal parts of the tibia was more solid and smooth with an apparent Codman’s triangle. The medial and caudal regions of the leg showed a soft tissue swelling together with an enlarged popliteal lymph node. The bony changes were compatible with an osteomyelitis or a neoplasia.

## Results

At presentation at the cardiology service of the authors’ institution the dog was bright, alert and responsive with a body condition score of 5 out of 9. The respiratory rate was 28 breaths/min and its type was costo-abdominal. The femoral pulse was powerful, regular, symmetric with a frequency of 108 bpm, without a pulse deficit. The rectal temperature was 38.8 °C. The coat, skin and the palpable lymph nodes showed no abnormalities, except for mildly enlarged popliteal lymph nodes. Mucous membranes were pink with a capillary refill time within 1 s. No signs of respiratory distress were noted. Cough could not be elicited with tracheal palpation. Auscultation of the lungs revealed no abnormalities. However, a grade 3 out of 6 continuous cardiac murmur was auscultated with the point of maximal intensity at the region of the left heart base. On the medial surface of the tibia hard irregularities were palpable.

For further assessment of the heart murmur transthoracic echocardiography was performed, which confirmed the presence of the clinically suspected left to right shunting patent ductus arteriosus (PDA). The pulmonary trunk had normal dimensions (pulmonary trunk to aortic ratio of 0.9; reference: 0.80–1.15) and no adult heartworms were seen in the trunk or in the right and left pulmonic arteries [1, 16–18]. The right atrial and right ventricular lumen- and wall dimensions appeared subjectively normal. The left ventricular diastolic and systolic lumen dimensions were within the reference ranges with a normalized diastolic left ventricular internal diameter of 1.79 (reference: 1.27–1.85), and a normalized systolic left ventricular internal diameter of 1.07 (reference: 0.71–1.26) measured on right parasternal cross-sectional M-mode images [19]. The left atrium was of normal size with a left atrium to aortic ratio of 1.3 (reference ≤ 1.6) measured on 2-dimensional right parasternal cross-sectional images [17, 20]. Mild mitral and aortic valve regurgitation jets were noted. The blood flow velocity in the aorta was mildly increased (2.5 m/s; reference < 2.0 m/s) [17, 21]. Simultaneous ECG showed a sinus arrhythmia with occasional solitary uniform ventricular premature complexes with right bundle branch block configuration and a short run of ventricular tachycardia.

The antigen test for *D. immitis* was repeated in two different additional laboratories using different test kits, and a blood sample for PCR evaluation of the microfilariae was submitted. Based on the positive blood antigen and positive blood microfilaria test results, the treatment protocol of the American Heartworm Society was started with oral doxycycline therapy (10.6 mg/kg BID) while the additional laboratory results were pending. Doxycycline therapy is also reported to be effective for the *Hepatozoon* co-infection [22].

The plasma concentration of N-terminal B-type natriuretic peptide (NTproBNP) was measured for further evaluation of the hemodynamic effect of the left to right shunting PDA [23]. The concentration was, at 387 pmol/l, in the lower part of the reference range [23]. The heartworm antigen test was repeated in two different additional veterinary laboratories using different test kits. The second laboratory confirmed the result of the first laboratory (initially negative and after heat treatment positive) using the IDEXX SNAP HTWM test kit (IDEXX Europe B.V.); the third laboratory, however, had a negative result using the WITNESS® Heartworm Antigen test kit (Zoetis, Parsippany-Troy Hills, New Jersey, USA) and was unable to perform heat treatment of the sample. Real-time PCR examination for circulating microfilariae was positive for *A. dracunculoides*, and negative for *D. repens*, *D. immitis* and *A. reconditum* (IDEXX Europe B.V.).

After a week of twice daily attempted oral doxycycline administration, the therapy had to be stopped because it caused anorexia, vomiting and lethargy, despite the fact that the referring veterinarian tried several possibilities to adjust the feeding regime of the dog (e.g. administration of the tablets with food or 2 h after feeding). After ceasing the oral doxycycline therapy, the gastrointestinal signs resolved spontaneously within a couple of days.

Based on the additional blood test results (PCR on microfilaria) a *D. immitis* (co)infection seemed unlikely and it was concluded that the microfilariae belonged to the *A. dracunculoides* species and the heat treatment of the blood samples led repeatedly to a false positive antigen test result for heartworm infection. Therefore, the protocol of adulticide therapy was stopped and empirical therapy was started for both the larval and the adult stadia of *A. dracunculoides*. One week after the last oral doxycycline administration, moxidectin spot-on therapy was started using two pipets, one for middle sized dogs (4–10 kg) and one for large dogs (10–25 kg), resulting in a total dose of 350 mg imidacloprid and 87.5 mg moxidectin (Advocate spot-on, Bayer Animal Health GmbH, Leverkusen, Germany). This spot-on therapy was repeated two more times at monthly intervals. One month after the third moxidectin administration a blood testing was negative for circulating microfilariae (native blood smear and Knott’s test). The antigen test also became negative, even after heat treatment. Additionally, the serum total protein, albumin and globulin concentrations were within the reference ranges. Two imidocarb dipropionate injections (5.4 mg/kg, SC, Carbesia, MSD Animal Health, Boxmeer, the Netherlands) were administered at 2-week intervals for the *Hepatozoon* infection; the first injection was administered simultaneously with the second moxidectin spot-on.

## Discussion

In the present report we describe a case where a blood sample tested using an antigen test for *D. immitis* was initially negative, but it turned to false positive following heat treatment. The suspicion of false positivity was raised when the real-time PCR revealed only microfilariae of *A. dracunculoides*, but not those of *D. immitis*. The fact that both antigen and microfilaria tests became negative after three monthly moxidectin spot-on treatments is indirect proof that the dog was infected solely with *A. dracunculoides* and not with (additional) *D. immitis*. *Hepatozoon* co-infection was a further clue to support this suspicion, as *Hepatozoon* and *A. dracunculoides* are both causing canine vector-borne diseases and both have the same intermediate host, the brown tick (*Rhipicephalus sanguineus*) [22, 24]. Though a slow kill protocol for the eradication of *D. immitis* consists of doxycycline and moxidectin, doxycycline should be administered for four weeks followed by monthly moxidectin administration for nine months to eradicate heartworms [25]. Because of gastrointestinal adverse effects, doxycycline therapy was ceased in the present dog within a week. Therefore, it is very unlikely that the three administrations of moxidectin spot-on alone had killed all adult heartworms and led to the test results becoming negative three months after the first administration of moxidectin spot-on. In cases of heartworm infection, the antigen test becomes negative only nine months after the completion of adulticide therapy [1].

*Acanthocheilonema dracunculoides* (syn. *Dipetalonema dracunculoides*) is a prevalent nematode in Spain and it is thought to have no clinical importance in dogs [26]. The adult *A. dracunculoides* live in the peritoneal and thoracic cavities of the canine hosts and they do not generally cause clinical signs [15, 26]. Although pharmacological studies about effective therapy for their adult examples are lacking, administration of monthly moxidectin spot-on at three occasions seemed to be sufficient to eradicate these parasites, shown by turning the initially positive antigen test for *D. immitis* after heat treatment to negative. Unlike the heartworm, which infects dogs by a mosquito bite, *A. dracunculoides* causes infection after ingestion of a brown tick, the worm’s intermediate host, when the dogs groom themselves or eat prey parasitized by an infected tick. Ingesting an infected tick is also the route of infection with *Hepatozoon* in dogs [22].

In the reported dog a left to right shunting PDA was found as a coincidental finding. Because the low plasma concentration of NTproBNP and the lack of left ventricular eccentric hypertrophy, no occlusion of this congenital cardiovascular anomaly was recommended in this already middle-aged dog [23].

The clinical and radiographic abnormalities of the left tibia could be the result of an osteomyelitis, which can be a manifestation of *Leishmania* or *Hepatozoon* infections [22, 27]. To prove this suspicion and to differentiate the lesion from other inflammatory and neoplastic lesions, a bone biopsy should have been performed. The owner declined this diagnostic test. Although microscopic differentiation of gamonts of *Hepatozoon canis* and *H. americanum* in a blood smear is impossible, the present dog was most likely infected with *H. canis*, as it is very unlikely that a Spanish stray dog had ever been to endemic areas of *H. americanum*. Musculoskeletal pathology is a rarely reported feature of *H. canis* infection, whereas it is common in dogs infected with *H. americanum*. In the present case, *Hepatozoon* infection was thought to be a clinically irrelevant coincidental finding based on the low number of infected neutrophils in the blood smear [22].

The ventricular premature complexes were thought to be coincidental findings in this dog too. Echocardiography did not reveal any possible cause of this arrhythmia. A myocarditis might have been present as both *Leishmania* and *Hepatozoon* infections are known to cause myocarditis [22, 28]. Measuring serum troponine-I concentration might have helped to prove this suspicion, but the owner declined this test. To evaluate the presence of potentially life-threatening ventricular tachyarrhythmias, a Holter ECG should have been performed. The owner chose not to perform this test either.

## Conclusions

We conclude that heat treatment of a blood sample with an initially negative antigen test for *D. immitis* can lead to a false positive result if the dog is infected with *Acanthocheilonema dracunculoides* (syn. *Dipetalonema dracunculoides*)*.* For this reason, real-time PCR is highly recommended in microfilaremic dogs to identify the nematode species present. Heat treatment should not be used routinely in geographic regions where additional microfilaria-producing canine-pathogenic nematodes, other than *D. immitis*, are endemic [29].

## Data Availability

Not applicable.

## Abbreviations

ELISA: Enzyme-linked immunosorbent assay
NTproBNP: N-terminal pro B-type natriuretic peptide
PCR: Polymerase chain reaction
PDA: Patent ductus arteriosus

## References

[1] American Heartworm Society. https://www.heartwormsociety.org. Accessed 14 Oct 2019.

[2] Starkey LA, Bowles JV, Payton ME, Blagburn BL (2017). Comparative evaluation of commercially available point-of-care heartworm antigen tests using well-characterized canine plasma samples. Parasit Vectors.

[3] Henry LG, Brunson KJ, Walden HS, Wenzlow N, Beachboard SE, Barr KL (2018). Comparison of six commercial antigen kits for detection of *Dirofilaria immitis* infections in canines with necropsy-confirmed heartworm status. Vet Parasitol.

[4] Gillis JM, Smith RD, Todd KS (1984). Diagnostic criteria for an enzyme-linked immunosorbent assay for occult heartworm disease: standardization of the test system in naturally exposed dogs. Am J Vet Res.

[5] Schnyder M, Deplazes P (2012). Cross-reactions of sera from dogs infected with *Angiostrongylus vasorum* in commercially available *Dirofilaria immitis* test kits. Parasit Vectors.

[6] Aroch I, Rojas A, Slon P, Lavy E, Segev G, Baneth G (2015). Serological crossreactivity of three commercial in-house immunoassays for detection of *Dirofilaria immitis* antigens with *Spirocerca lupi* in dogs with benign esophageal spirocercosis. Vet Parasitol.

[7] Krucik DD, Van Bonn W, Johnson SP (2016). Association between positive canine heartworm (*Dirofilaria immitis*) antigen results and presence of *Acanthocheilonema odendhali* microfilaria in California sea lions (*Zalophus californianus*). J Zoo Wildl Med.

[8] Little S, Saleh M, Wohltjen M, Nagamori Y (2018). Prime detection of *Dirofilaria immitis*: understanding the influence of blocked antigen on heartworm test performance. Parasit Vectors.

[9] Ciucă L, Genchi M, Kramer L, Mangia C, Miron LD, Del Prete L (2016). Heat treatment of serum samples from stray dogs naturally exposed to *Dirofilaria immitis* and *Dirofilaria repens* in Romania. Vet Parasitol.

[10] Venco L, Manzocchi S, Genchi M, Kramer LH (2017). Heat treatment and false-positive heartworm antigen testing in *ex vivo* parasites and dogs naturally infected by *Dirofilaria repens* and *Angiostrongylus vasorum*. Parasit Vectors.

[11] Ortega-Mora LM, Gómez-Bautista M, Rojo-Vázquez FA (1989). The acid phosphatase activity and morphological characteristics of *Dipetalonema dracunculoides* (Cobbold, 1870) microfilariae. Vet Parasitol.

[12] Peribáñez MA, Lucientes J, Arce S, Morales M, Casillo JA, Gracia MJ (2001). Histochemical differentiation of *Dirofilaria immitis*, *Dirofilaria repens* and *Acanthocheilonema dracunculoides* microfilariae by staining with a commercial kit. Vet Parasitol.

[13] Magnis J, Lorentz S, Guardone L, Grimm F, Magi M, Naucke TJ (2013). Morphometric analyses of canine blood microfilariae isolated by the Knott's test enables *Dirofilaria immitis* and *D. repens* species-specific and Acanthocheilonema (syn.Dipetalonema) genus-specific diagnosis. Parasit Vectors..

[14] Rishniw M, Barr SC, Simpson KW, Frongillo MF, Franz M, Alpizar JLD (2006). Discrimination between six species of canine microfilariae by a single polymerase chain reaction. Vet Parasitol.

[15] Muñoz C, Gonzálvez M, Rojas A, Martínez-Carrasco C, Baneth G, Berriatua E (2020). Massive microfilaremia in a dog subclinically infected with *Acanthocheilonema dracunculoides*. Parasitol Int.

[16] Thomas WP, Gaber CE, Jacobs GJ, Kaplan PM, Lombard CW, Moise NS (1993). Recommendations for standards in transthoracic two-dimensional echocardiography in the dog and cat Echocardiography Committee of the Specialty of Cardiology American College of Veterinary Internal Medicine. J Vet Intern Med..

[17] Boon J (2010). Veterinary echocardiography.

[18] Serres F, Chetboul V, Gouni V, Tissier R, Sampedrano CC, Pouchelon JL (2007). Diagnostic value of echo-Doppler and tissue Doppler imaging in dogs with pulmonary arterial hypertension. J Vet Intern Med.

[19] Cornell CC, Kittleson MD, Della Torre P, Häggström J, Lombard CW, Pedersen HD (2004). Allometric scaling of M-mode cardiac measurements in normal adult dogs. J Vet Intern Med.

[20] Hansson K, Häggström J, Kvart C, Lord P (2002). Left atrial to aortic root indices using two-dimensional and M-mode echocardiography in Cavalier King Charles spaniels with and without left atrial enlargement. Vet Radiol Ultrasound.

[21] Yuill CDM, O’Grady MR (1991). Doppler-derived velocity of blood flow across the cardiac valves in the normal dog. Can J Vet Res.

[22] Baneth G (2011). Perspectives on canine and feline hepatozoonosis. Vet Parasitol.

[23] Marinus SM, van Engelen H, Szatmári V (2017). N-terminal pro-B-type natriuretic peptide and phonocardiography in differentiating innocent cardiac murmurs from congenital cardiac anomalies in asymptomatic puppies. J Vet Intern Med.

[24] Olmeda-García AS, Rodríguez-Rodríguez JA, Rojo-Vázquez FA (1993). Experimental transmission of *Dipetalonema dracunculoides* (Cobbold, 1870) by *Rhipicephalus sanguineus* (Latreille, 1806). Vet Parasitol.

[25] Genchi M, Vismarra A, Lucchetti C, Viglietti A, Crosara S, Gnudi G (2019). Efficacy of imidacloprid 10%/moxidectin 2.5% spot on (Advocate®, Advantage Multi®) and doxycycline for the treatment of natural Dirofilaria immitis infections in dogs. Vet Parasitol..

[26] Bolio ME, Montes AM, Alonso FD, Gutiérrez C, Bernal LJ, Rodríguez-Vivas RI (2004). Prevalence of *Dipetalonema dracunculoides* in dogs in Murcia. Spain Vet Rec.

[27] de Souza AI, Juliano RS, Gomes TS, de Araujo DS, Borges AM, Tafuri WL (2005). Osteolytic osteomyelitis associated with visceral leishmaniasis in a dog. Vet Parasitol.

[28] Martínez-Hernández L, Casamian-Sorrosal D, Barrera-Chacón R, Cuesta-Gerveno JM, Belinchón-Lorenzo S, Gómez Nieto LC (2017). Comparison of myocardial damage among dogs at different stages of clinical leishmaniasis and dogs with idiopathic chronic kidney disease. Vet J.

[29] European Society of Dirofilariosis and Angiostrongylosis. https://www.esda.vet. Accessed 08 Apr 2020.

